# Identifying current driver rehabilitation practices for clients with physical impairments

**DOI:** 10.1177/03080226211067432

**Published:** 2022-02-10

**Authors:** Brittany Langereis, Sarah Semeniuk, Lisa Kristalovich, W.Ben Mortenson

**Affiliations:** 1 Department of Occupational Science and Occupational Therapy, Faculty of Medicine, 8166 University of British Columbia, Vancouver, BC, Canada; 2 GF Strong Rehabilitation Centre, Vancouver, BC, Canada; 3 International Collaboration on Repair Discoveries, Vancouver, BC, Canada

**Keywords:** Adaptive driving equipment, automobile driving, driver rehabilitation, physical impairments, transportation, occupational therapy

## Abstract

**Introduction:**

Driving is an important occupation that influences a person’s ability to access community resources and participate in meaningful activities. Occupational therapists and other clinicians who are trained as driver rehabilitation specialists provide interventions to improve function and independence in driving. The objective of this study was to examine consistency of adaptive driving equipment prescription and driver retraining recommendations for clients with physical impairment and identify factors that influence driver rehabilitation recommendations.

**Methods:**

Clinicians practicing in Canada and the United States were surveyed electronically. Data were analyzed using descriptive statistics and content analysis.

**Results:**

Out of 36 respondents, the majority (97.2%) were occupational therapists. Completion of in-clinic (97.0%) and on-road (84.4%) evaluations were consistent among the majority of respondents, yet components comprising evaluations varied. Fewer respondents were in agreement in equipment recommendations for secondary controls. Respondents emphasized the importance of clinical reasoning skills when making equipment and intervention recommendations.

**Conclusion:**

Further development of clinical guidelines and advanced training in driver rehabilitation may be beneficial to facilitate clinical reasoning and improve consistency of practice.

## Introduction

Driving a motor vehicle is a meaningful occupation many people value as a means to access resources, participate in work, and engage in the community. (Davis et al., 2016; Ross et al., 2018). When an individual experiences physical impairment from an injury or illness, their ability to operate driving controls may change and this may prevent them from driving an un-adapted vehicle safely and independently. This can negatively impact a person’s sense of identity, autonomy, and quality of life (Oxley & Whelan, 2008), lead to feelings of isolation and depression, and cause decreased participation in valued life roles (Cammarata et al., 2017; Davis et al., 2016). Resuming driving post-injury or illness is recognized as an important sign of recovery for many individuals (Čižman et al., 2017; Ross et al., 2018; Stolwyk et al., 2019). Approximately 50% of stroke survivors “prioritize it as the most important goal” of their rehabilitation (Cammarata et al., 2017: 23).

Individuals with a medical condition must “seek clearance from a licensing jurisdiction to learn, or continue to drive” and are usually referred by a medical practitioner to driver rehabilitation services in order to return to driving (Unsworth & Baker, 2014). Some individuals require vehicle modification and/or adaptive driving equipment to enable safe return to driving. Adaptive driving equipment can include low- or high-tech options to assist clients in safely operating and accessing steering, acceleration, and brake function (e.g., pedal extension, steering wheel aids) (Di Stefano et al., 2019).

Two previous surveys have found high variability in driver rehabilitation specialists’ (DRS) training practices for clients returning to driving using hand controls in the United States (Lowe et al., 2014) and the choice of assessment tools, recommendations, and decision-making regarding driving evaluations for individuals with physical impairments among Canadian DRS (Vrkljan et al., 2015). Since then, the Association for Driver Rehabilitation Specialists (ADED) has published the *Best Practice Guideline for Delivery of Driver Rehabilitation Services* (2016) which provides detailed recommendations for evaluations but has limited guidance on prescription and training of adaptive driving equipment.

Therefore, this study was conducted in Canada and the United States of America to collect more contemporary information about the consistency of current driving practice recommendations and interventions for individuals with physical impairment who require adaptive driving equipment. More specifically, this survey aimed to:

1. examine the consistency of practice in driver rehabilitation training and interventions
2. investigate the factors that influence a clinician’s decision-making process for equipment and intervention recommendations.

## Method

A web-based survey was used to collect quantitative and qualitative data related to the driver rehabilitation practice process. The Checklist for Reporting Results of Internet E-Surveys (CHERRIES) was used to report on the web-based survey (Eysenbach, 2004). The study was approved by the University of British Columbia’s Behavior Research Ethics Board in 2019 (Approval #: H19-01536).

### Survey questionnaire

The survey included both close-ended questions (nominal and ordinal format) and open-ended questions. The survey comprised three distinct sections. Section 1 collected demographic data related to clinical background and experience, professional education, and province/state of practice. Section 2 elicited information about respondents’ driver rehabilitation practice processes (i.e., use of practice protocols, factors that influence training recommendations, and driving behaviors that indicate safe discharge from driver rehabilitation services). For example, respondents were asked: “When conducting an initial evaluation of a client with physical impairment, how frequently do you typically complete an in-clinic evaluation?” Section 3 included two case studies to explore variations in clinical practice in specific situations involving clients with a specific physical impairment. For example, for each case study, they were asked to indicate “What equipment would you typically recommend for this client? Check all that apply.” They also had the option to add additional equipment not listed. To increase consistency of question interpretation, respondents were directed to consider their practice process for individuals with physical impairment, excluding those individuals with mixed physical, cognitive, and/or affective impairment.

### Recruitment process

Participants included clinicians currently practicing in driver rehabilitation who understood English. Participants were recruited from a variety of online platforms including the Canadian Association of Occupational Therapists (CAOT) Research Listing and two international Facebook groups for driver rehabilitation specialists. Snowball sampling was utilized as participants were encouraged to share the survey with colleagues. The survey was open for participation for 9 weeks. Written informed consent was obtained from all participants.

### Data management and analysis

Thirty-six surveys were included in the analysis. Of these, 86.8% were partially completed ranging from 94.7% to 18.4% complete. An additional 18 surveys were submitted but excluded from the analysis as respondents did not indicate they were currently practicing in driver rehabilitation and the study objective was to examine current clinical practice. The average proportion of survey completion was 78.7%. Descriptive statistics were used to summarize all quantitative data. Data collected from open-ended question responses were analyzed using directed content analysis (Hsieh & Shannon, 2005). With directed content analysis, researchers use existing theory or priori ideas when developing the initial codes. In this case, the Person, Environment, Occupation (PEO) Model was used to develop the initial coding scheme, which was modified and refined as the analysis progressed. To strengthen the credibility of qualitative research, coding was completed by the first two authors independently and later reviewed together by all members of the research team to corroborate the evolving codes (Carpenter and Suto, 2008). All initial codes were then organized into themes and reviewed by all four authors to ensure their relevance.

## Results

Table 1 provides an overview of respondent demographics. The majority of respondents were occupational therapists (97.2%) and many identified as a DRS (33.3%) or Certified Driver Rehabilitation Specialist (CDRS) (52.7%). The only respondent who was not an OT identified as a DRS and driver instructor. Driver rehabilitation training was most frequently completed through workshops or conferences and peer mentoring.

**Table 1.** Demographic information and other characteristics of survey respondents.

Characteristics	*N* (%)
Location of practice, *n* = 36	
Canada	17 (47.2)
United States	18 (50.0)
Unspecified	1 (2.8)
Professional background^a^, *n* = 36	
Occupational therapist	35 (97.2)
Physiotherapist	1 (2.8)
Driver rehabilitation specialist	12 (33.3)
Certified driver rehabilitation specialist	19 (52.7)
Driver instructor	8 (22.2)
Driver rehabilitation training^a^, *n* = 36	
Driver rehabilitation workshop or conference	32 (88.9)
American occupational therapy association specialty certification in driving and community mobility	2 (5.6)
McGill graduate certificate in assessing driving Capability	2 (5.6)
Association of driver rehabilitation specialists conferences	26 (72.2)
Peer mentoring	28 (77.8)
Other	7 (19.4)
Years of experience in driver rehabilitation, *n* = 35	
1–3 years	12 (34.3)
4–6 years	5 (14.3)
7–9 years	2 (5.7)
10–12 years	1 (2.9)
13+ years	15 (42.9)
Number of hours spent working in driver rehabilitation per week, *n* = 33	
1–4 h	6 (18.2)
5–9 h	3 (9.1)
10–14 h	5 (15.2)
15–19 h	3 (9.1)
20+ hours	16 (48.5)

^a^Respondents were able to choose more than one option when responding to this question.

Table 2 describes evaluation methods, including specific on-road maneuvers, environments, and conditions that clients are expected to demonstrate competence in prior to discharge. The majority of respondents always completed an in-clinic evaluation (97%) and an on-road evaluation (84.4%), but completion of a stationary in-vehicle evaluation (67.7%) or final fitting (77.7%) was less consistently completed as part of the typical evaluation process. During open-ended questions, respondents indicated the decision depended on the client situation but did not provide specific factors contributing to the selection of evaluation methods. Prompts for specific client indicators were not included in this survey.

**Table 2.** Evaluation methods, on-road maneuvers, and driving environments considered essential for clients with adaptive driving equipment.

Characteristics	*N* (%)
Evaluation methods	
Initial assessment components, *n* = 33	
In-clinic evaluation (always completed)	32 (97.0)
On-road evaluation (always completed)	27 (84.4)
Stationary in-vehicle evaluation (always completed)	21 (67.7)
Final fitting, *n* = 28	
Completed with all clients	10 (35.7)
Completed with some clients	14 (50.0)
completed	4 (14.2)
Provincial/state driving licensing exam required, *n* = 28	
Yes	12 (42.9)
No	16 (57.1)
On-road maneuvers^a^, *n* = 28	
Gradual, controlled acceleration	27 (96.4)
Gradual, controlled braking	28 (100)
No error with choosing correct pedal	27 (96.4)
Use of secondary vehicle controls (i.e., turn signal, windshield wipers, horn) while in traffic	27 (100)
Lane changes	28 (100)
Forward stall parking	23 (88.5)
Reverse stall parking	15 (57.7)
Parallel parking	6 (26.1)
Driving environments, *n* = 29	
Residential roads	29 (100)
Main roads	29 (100)
Intersections	28 (96.6)
City driving	28 (96.6)
Park/school zones	25 (86.2)
Highway driving	19 (65.5)
Hills	16 (55.1)
Roundabouts	9 (31.0)
Cyclist lanes	6 (20.6)
Winter conditions	6 (20.6)
Rain	2 (6.9)

^a^Respondents were able to choose more than one option when responding to this question.

When discharging a client from driver rehabilitation services, 29 out of 30 of those who responded to this question (96.7%) decided based on their observation of the client, 17 out of 22 (77.3%) who responded to this question decided based on consultation with the driver instructor, and 4 out of 15 (26.7%) that responded to this question reported that the driver instructor made this decision. Of the 12 respondents out of 28 (42.9%) that indicated clients were required to pass a provincial/state driving licensing exam, four were practicing in Canada and eight were practicing in the United States.

With case studies, over three-quarters of those who responded to those questions recommended the same adaptive driving equipment for primary vehicle controls (steering, acceleration and braking) for a specific client presentation. In the first case study with a client with right hemiparesis, 78.3% of the respondents recommended either a multi-functional spinner or basic spinner for steering, 75% recommended an electronic foot accelerator with pedal guard, and 88.2% did not recommend hand controls. The second case study was a client with a thoracic level spinal cord injury and paraplegia. In this case study, 91.7% recommended a basic spinner for steering, and 85.7% recommended mechanical hand controls. There was less agreement between respondents on secondary control adaptive driving equipment (turn signals, windshield wipers, etc.). For the first case study, 30.0% recommended a turn signal extension and 43.8% recommended switch-activated secondary controls. For second case study, 35.3% recommended a turn signal extension and 31.3% recommended switch-activated secondary controls.

**Figure 1.**
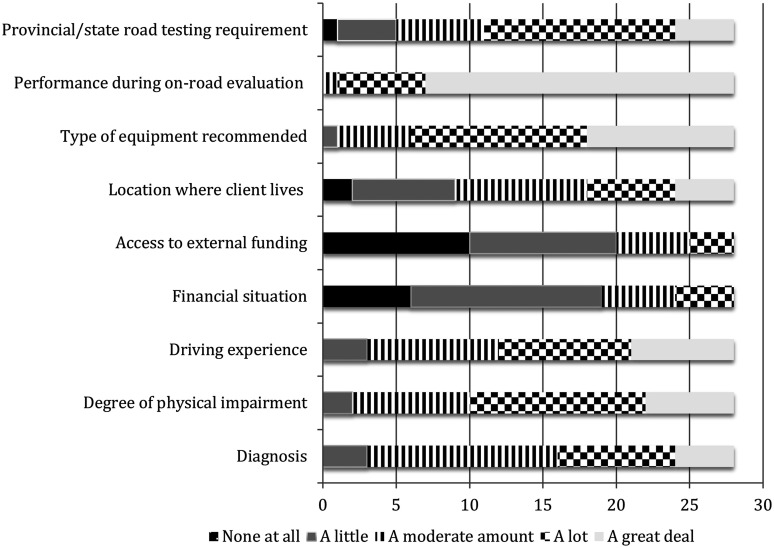
Factors impacting the number of driver retraining hours recommended for clients with physical impairments (*n* = 28).

A variety of factors influenced the number of driver retraining hours recommended (Figure 1). The most significant was the “client’s performance during the on-road evaluation.” Other factors that were strongly weighted included the “type of equipment that is recommended” and “client’s degree of physical impairment.” Factors with little to no impact on recommendations were “a client’s access to external funding” and “client’s financial situation.” Twenty respondents (62.5%) recommended a minimum number of hours of driver retraining for clients using adaptive driving equipment.

**Figure 2.**
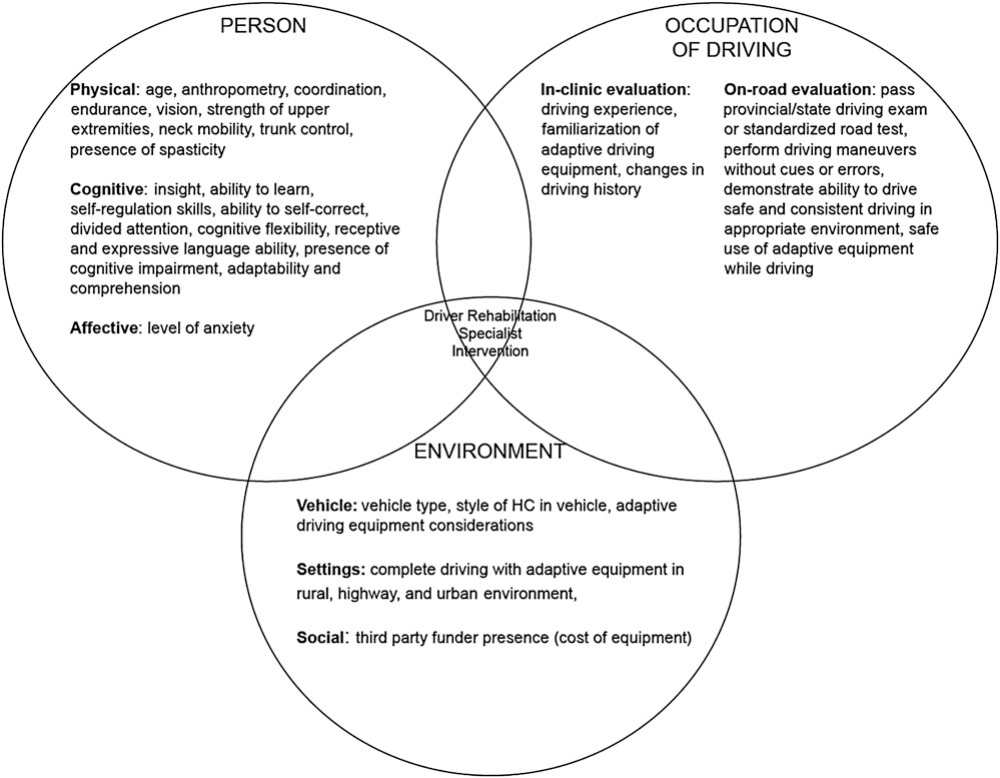
Factors that influence clinician’s clinical reasoning and decision making in driver rehabilitation practice.

In response to open-ended questions, respondents placed an emphasis on a client-centered approach, providing responses such as “there is no ‘typical’ client. It all depends” and driver rehabilitation “is very person centered, there is not a formula you can use.” As illustrated in Figure 2, responses were grouped into themes that aligned with the PEO model, a conceptual model used in OT practice. The “Person” category encapsulated the client’s attributes that affect clinical reasoning and subsequent recommendations. While the survey directed respondents to consider clients with only physical impairment, respondents used a holistic approach and most frequently noted cognitive attributes such as “insight” and “ability to learn” as factors contributing to clinical reasoning. Within “Occupation of Driving,” all performance factors directly related to or involving driving were sorted into sub-categories based on whether they would typically be assessed during an in-clinic or on-road evaluation. Many respondents identified “safe” driving behaviors as an indicator of competence. They described looking for “safe driving with the equipment,” the ability to “drive in various environments safely,” and the ability to “safely complete all maneuvers.” Although it was mentioned 13 times, none of the respondents provided a concrete example or clear description of the concept of “safe/safety.” To measure a client’s overall competence objectively, many respondents utilized “standardized” road tests. For some, an “error free” driving performance was required for successful completion of road test evaluations. Last, the “Environment” category refers to additional factors that may affect equipment and intervention recommendations, such as the client’s driving environment, vehicle details, and access to funding.

## Discussion

This study provides insights into consistency of practice in driver rehabilitation. The majority of respondents conducted in-clinic and on-road evaluations when determining a client’s fitness to drive, which is congruent with existing recommendations (Association for Driver Rehabilitation Specialists, 2016; Canadian Association of Occupational Therapists, n.d; Di Stefano et al., 2019) and other empirical studies (Cammarata et al., 2017; Vrkljan et al., 2015). Specific components of the in-clinic evaluation found to be consistent among respondents aligned with recommendations within ADED’s *Best Practice Guidelines* (2016). These included reviewing clients’ medical and driving histories and performing in-clinic evaluations of physical and cognitive components such as coordination, strength, and executive function.

On-road evaluations are important to assess the impact of the client’s function and cognition on their “actual driving behavior and driving potential” (Association for Driver Rehabilitation Specialists, 2016: 18), as they provide insight into a client’s current driving behaviors and aid in the development of more individualized driver retraining recommendations (Kowalski et al., 2010). Classen et al. (2016) identified that on-road evaluation is important in determining a client’s fitness to drive, but specific components of on-road evaluations may differ among service providers. In this survey, respondents consistently used a standardized road test to determine a client’s readiness to return to driving. Not surprisingly, a client’s performance during an on-road evaluation had the largest impact on clinical reasoning for recommendations.

Although there is consistency among survey results and the literature regarding the importance and utility of on-road evaluation, there is limited expert opinion on what defines an on-road evaluation (Dickerson, 2013) as no single approach or evaluation criteria is accepted as the “gold standard” (Kowalski et al., 2007). This will likely depend on the intended purpose of the evaluation (Kowalski et al., 2010). This was evident with multiple respondents identifying “safe” as an evaluation criteria. Driving is an activity of daily living where the person’s actions may place themselves or other road users at risk of injury. Accordingly, the evaluation criteria of “safe” appear appropriate but is subjective and has not been operationalized.

Overall, evaluation practices employed within driver rehabilitation appear to be consistent between clinicians, but there is some variability in practice. Respondents were not asked to provide clinical reasoning for their recommendations and thus, the rationale behind the different recommendations cannot be determined. Based on the PEO model, it appears that clinicians adapted their recommendations based on the case scenarios. The clinicians may have considered potential issues related to cognitive and affective domains, even though the person was being evaluated for a physical impairment. This multi-factoral approach may suggest that a rigid prescription structure is not suitable for this area of practice.

Clients’ financial situation and access to external funding had little to no influence on driver retraining recommendations, aligning with the principles and professional responsibilities of driver rehabilitation practice. As recommendations for equipment prescriptions do not appear to change based on funding, client care does not appear to be compromised based on lack of funding. However, the literature has identified lack of funding for adaptive equipment prescription as a potential barrier to implementation (Di Stefano et al., 2019).

Driver rehabilitation requires clinicians to navigate both healthcare and driver licensing systems. Respondents in some geographic areas indicated a government driver licensing examination was required for an individual to use adaptive driving equipment and respondents in other areas indicated a government driver licensing examination was not required. These variations in government driver licensing requirements likely contribute to variability in driver rehabilitation practice.

While this survey identified consistent adaptive driving equipment recommendations between clinicians, these findings also identified inconsistency in equipment recommendations for the operation of secondary controls. There are multiple possible reasons for this inconsistency. The case study was brief and provided a general description, such as hemiparesis. Physical impairments have a range of impairment and respondents may have interpreted the person’s abilities differently within the given description. It is possible the same adaptive driving equipment may not have been available to clinicians in different geographic areas or clinicians may not have been aware of the full range of equipment available. Different equipment may alter the ease of use of the essential vehicle controls and alter equipment cost.

Defined as the thinking process of “planning, conducting and reflecting” on one’s practice (Armstrong et al., 2020: 78), clinical reasoning is an integral component of occupational therapy practice. Respondents and the literature are in agreement that a formulaic approach (e.g., clinical pathway) for equipment or intervention recommendations is inappropriate because of the need for client-specific clinical reasoning (Association for Driver Rehabilitation Specialists, 2016; Di Stefano et al., 2019). This highlights the complexity of driver rehabilitation practice and supports the need for clinicians to have advanced training to identify individual client characteristics and factors that impact clinical reasoning and subsequent recommendations.

### Limitations

One major limitation of this study was its sample size. In order to increase the generalizability of the findings, a larger and more representative sample would be beneficial, although it should be noted that only a relatively small number of therapists practice in this area. In addition, interviews would have been helpful to interpret the survey findings. The study design did not permit in-depth exploration of the clinical reasoning behind specific decisions.

### Future research

Further research could explore the clinical reasoning and specific factors contributing to equipment prescription and evaluation methods selected. Studies in countries other than Canada and the USA would increase the generalizability of findings and identify commonalities and differences in jurisdictional processes that may affect clinical reasoning. Given that “safety” as an indicator of driving competence is a subjective interpretation of driving performance, consensus-based approaches could be used to operationalize this term. Future studies could then evaluate if this helps to promote consistency when conducting evaluations. Although specialized education is recommended prior to working in driver rehabilitation (American Occupational Therapy Association, 2015; Canadian Association of Occupational Therapists, 2009; Davis et al., 2016), future research could determine whether the type of driver rehabilitation training influences the consistency of practice. The factors contributing to variations in evaluation components and equipment recommendations for secondary vehicle controls could be further investigated.

## Conclusion

This study examined various aspects of driver rehabilitation practice in Canada and the United States from clinicians’ perspectives. The findings add to the existing literature by identifying practices that driver rehabilitation clinicians frequently utilize. Consistencies and inconsistencies in practice were highlighted along with factors that influence clinicians’ clinical reasoning process when prescribing adaptive driving equipment and making recommendations for driver retraining. Driver rehabilitation specialist report using their clinical reasoning skills rather than adhering to rigid clinical pathways. Advanced education in driver rehabilitation and use of clinical guidelines and a theoretical model may assist clinicians in identifying appropriate factors to consider when providing driver rehabilitation services.

## Key findings

• Individualized driver rehabilitation plans are important.
• The majority of clinicians complete in-clinic and on-road evaluations of adaptive driving equipment as indicated.
• Primary vehicle control prescriptions and intervention recommendations are generally consistent.

## What the study has added

• As highlighted by the conceptual model, taking a client-centered approach and formulation of individualized plans for each client are important components of driver rehabilitation practice.
